# The Prevalence of *Helicobacter pylori* Infection in the Adult Population of Russia: A Systematic Review and Meta-Analysis

**DOI:** 10.3390/epidemiologia6030047

**Published:** 2025-08-12

**Authors:** Dmitrii N. Andreev, Alsu R. Khurmatullina, Igor V. Maev, Dmitry S. Bordin, Sayar R. Abdulkhakov, Yury A. Kucheryavyy, Petr A. Beliy, Filipp S. Sokolov

**Affiliations:** 1 Department of Internal Disease Propaedeutics and Gastroenterology, Russian University of Medicine, 127473 Moscow, Russia; 2 Department of Pancreatic, Biliary and Upper Digestive Tract Disorders, A. S. Loginov Moscow Clinical Scientific Center, 111123 Moscow, Russia; 3 Department of General Medical Practice and Family Medicine, Tver State Medical University, 170100 Tver, Russia; 4 Department of Internal Diseases, Institute of Fundamental Medicine and Biology, Kazan (Volga Region) Federal University, 420008 Kazan, Russia; 5 Department of Primary Care and General Practice, Kazan State Medical University, 420012 Kazan, Russia; 6 Ilyinskaya Hospital, 143421 Krasnogorsk, Russia; 7 Department of Pharmacology, Russian University of Medicine, 127473 Moscow, Russia

**Keywords:** *H. pylori*, epidemiology, prevalence, Russia, dynamics, eradication

## Abstract

**Objective:** The objective of this study is to assess the dynamics of *Helicobacter pylori* infection prevalence among adults in Russia. **Methods:** A systematic search was conducted in MEDLINE/PubMed, EMBASE, Cochrane, RSCI, and Google Scholar for studies published between 1985 and 27 February 2025, following PRISMA guidelines. The meta-analysis was registered in PROSPERO (CRD420251011643). **Results:** Twenty studies were included (n = 117,841; weighted mean age: 43.71 ± 16.23 years), all using validated diagnostic methods. The pooled prevalence from 1994 to 2024 was 62.847% (95% CI: 55.101–70.274), including 45.143% (95% CI: 41.390–48.923) by the ^13^C-urea breath test and 75.806% (95% CI: 64.213–85.742) by serology. Prevalence declined over time: it was 79.334% before 2005, 74.074% in 2006–2011, and 66.319% in 2012–2017, and it has been 42.949% since 2018. Meta-regression confirmed a significant decrease (coefficient: −3.773% per year, *p* < 0.001). **Conclusions:** A significant decline in the prevalence of *H. pylori* has been observed, however, it remains relatively high and requires continued efforts aimed at diagnosis and eradication.

## 1. Introduction

*Helicobacter pylori* are microaerophilic, spiral-shaped, Gram-negative bacteria that colonize the human gastric mucosa and are a leading causative factor in the development of a range of gastroduodenal diseases [1,2]. *H. pylori* consistently causes chronic gastritis, which can progress to severe complications such as peptic ulcer disease, MALT lymphoma, and gastric cancer (GC) [1]. Nearly 90% of GC cases are associated with *H. pylori* infection, which was classified as a Group 1 carcinogen by the World Health Organization (WHO) in 1994 [3,4]. In 2022, according to the International Agency for Research on Cancer (IARC) of the WHO, 968,784 new cases of GC were reported worldwide, resulting in 660,175 deaths [5].

Recent systematic reviews and meta-analyses indicate that over 40% of the global population is infected with *H. pylori* [6,7]. However, there has been a global decline in the prevalence of *H. pylori* infection, from 58.2% in the 1980–1990 period to 43.1% in 2011–2022 [8]. This trend is particularly evident in developed countries and populations actively implementing the “test-and-treat” strategy [8,9].

A similar trend has been observed in Russia, the largest country in the world with a population of over 145 million people, where a recent large multicenter study from 2017 to 2019 reported a decline in adult infection rates from 42.5% to 35.3% [10]. This positive trend has influenced the dynamics of GC incidence and mortality rates in Russia, although GC remains a significant medical and social problem in the country [11,12]. According to official federal data for 2023, GC was diagnosed in 19,380 patients and ranked as the fifth most common cause of oncological morbidity [11]. Moreover, regarding cancer-related mortality among men in Russia, GC ranks second (9.4%), while among women, it ranks fourth (7.4%) [11]. An accurate epidemiological assessment of *H. pylori* infection prevalence in the population is crucial, as the infection can remain asymptomatic for a long time while causing progressive changes in the gastric mucosa, thereby multiplying the risk of associated pathologies [13].

Data on the prevalence of *H. pylori* infection in Russia are limited, and no large meta-analytical studies systematizing the results of population-based research have been conducted. The objective assessment of *H. pylori* infection prevalence in the Russian population is complicated by the country’s vast size and multi-ethnic population. In some multicenter population-based studies, the prevalence of *H. pylori* infection ranged from 35% to 86% [10,14,15]. The primary objective of this meta-analysis was to systematize data on *H. pylori* infection prevalence among the adult population of Russia.

## 2. Materials and Methods

### 2.1. Study Sources and Search

This literature review was conducted following the PRISMA 2020 guidelines (File S1) [16] and was pre-registered in the PROSPERO database (CRD420251011643) to ensure methodological transparency. The search encompassed multiple academic databases, including MEDLINE/PubMed, EMBASE, Cochrane, the Russian Science Citation Index, and Google Scholar, covering studies published from 1 January 1985 to 27 February 2025. The search strategy in MEDLINE/PubMed included the following terms: “((“H. pylori” [MeSH Terms] OR “H. pylori” [Title/Abstract] OR “H. pylori” [Title/Abstract] OR “H. infections” [MeSH Terms] OR “H.” [Title/Abstract] OR “pylori” [Title/Abstract]) AND (“Russia” [MeSH Terms] OR “Russia” [Title/Abstract] OR “Russian” [Title/Abstract] OR “Siberia” [Title/Abstract] OR “Moscow” [Title/Abstract] OR “Saint Petersburg” [Title/Abstract] OR “Ural” [Title/Abstract] OR “Far East” [Title/Abstract])).”

### 2.2. Study Selection

The screening of retrieved records was performed in Rayyan, with A.R.K. and D.N.A. reviewing titles and abstracts, followed by a full-text assessment of potentially relevant studies. Reviewers screened the studies independently at both levels of screening. Studies were eligible for inclusion if they met the following criteria: published in English or Russian, contained detailed descriptive statistics (including the prevalence of *H. pylori*-positive and -negative individuals), focused on the adult population of Russia, and clearly described verified diagnostic methods for *H. pylori* infection. Exclusion criteria included studies involving participants with comorbidities that could independently influence *H. pylori* test results (e.g., chronic systemic inflammatory diseases, autoimmune gastrointestinal diseases), populations aged below 18 years, populations affected by environmental factors (e.g., radiation), and populations with diagnosed gastrointestinal diseases or symptoms (e.g., dyspepsia, peptic ulcer disease, inflammatory bowel disease). Additionally, research focusing on cancer patients was excluded to maintain specificity. In cases where data overlapped across multiple publications, only one study was retained for the final analysis. The quality of the included studies was assessed using the Newcastle–Ottawa scale (NOS) (File S2).

The risk of bias was assessed by two independent reviewers (F.S.S. and P.A.B.) using the Newcastle–Ottawa scale for observational studies. Inter-rater agreement was quantified using Cohen’s kappa statistic, with the following widely accepted interpretation scale: poor (κ ≤ 0.20), fair (κ = 0.21–0.40), moderate (κ = 0.41–0.60), good (κ = 0.61–0.80), and excellent agreement (κ > 0.80). The analysis encompassed both global inter-rater consistency and item-level agreement using these established thresholds.

### 2.3. Data Extraction

The extracted information comprised publication year, study location, *H. pylori* identification methods, total number of patients, and number of *H. pylori*-positive cases. Key study characteristics and outcome data were independently extracted by two authors (S.R.A. and D.S.B.). Whenever necessary, study protocols were identified, and corresponding authors were contacted for clarification or missing data. Disagreements were resolved through discussion or consultation with a third reviewer (Y.A.K.). If the study’s relevance remained uncertain, additional reviewers (P.A.B. and F.S.S.) participated in decision-making. This applies to both the study screening process and the data abstraction process.

### 2.4. Statistical Analysis

Statistical analysis was performed using MedCalc Statistical Software Program 23.0.6 (Ostend, Belgium) on Microsoft Windows 11 (Microsoft Corporation, Redmond, WA, USA). The overall frequency estimates of H. pylori prevalence in Russia population were reported with 95% confidence intervals (CIs). Study heterogeneity was evaluated using Cochrane’s Q test and I^2^ statistics, with significant heterogeneity defined as *p* < 0.05 and I^2^ > 75%. The prevalence of *H. pylori* infection in patients was pooled by a random-effects model. The dynamics of *H. pylori* infection through the years were evaluated by means of meta-regression with Python version 3.9.21 (Amsterdam, The Netherlands). To address substantial heterogeneity, a sensitivity analysis was conducted: first, the proportion of *H. pylori* patients in studies with an NOS score below 7 was evaluated; second, the proportion of these patients in studies with an NOS score of 7 or higher was assessed. Potential publication bias was evaluated using a funnel plot, Begg–Mazumdar’s correlation test, and Egger’s test.

## 3. Results

### 3.1. Search Results

**Figure 1:**
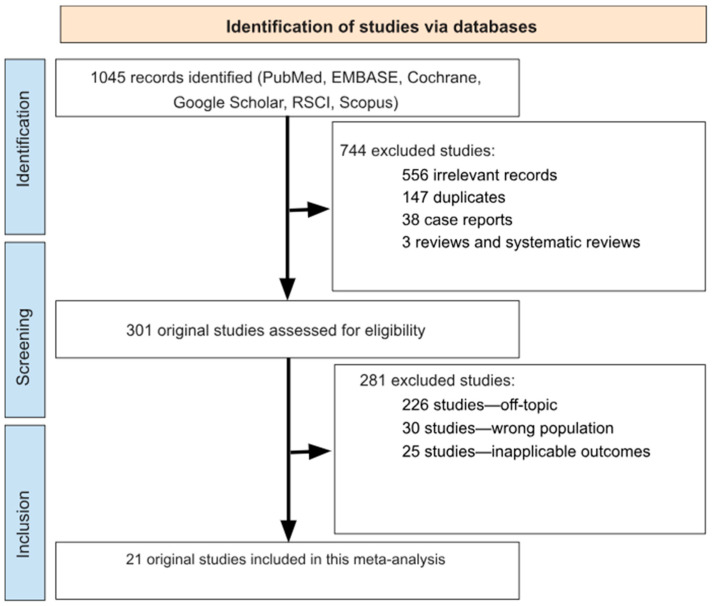
Flow chart detailing study selection strategy.

A total of 1045 studies were retrieved for initial screening. Of these, 744 were excluded for not meeting the inclusion criteria (556 irrelevant, 147 duplicates, 38 case reports, 3 reviews and systematic reviews). After evaluating 301 studies in detail, an additional 281 were excluded due to topic irrelevance (226 studies), inappropriate population (30 studies), or unsuitable outcomes (25 studies) (Figure 1). Ultimately, 20 studies were included in the meta-analysis (Table 1) [10,15,17,18,19,20,21,22,23,24,25,26,27,28,29,30,31,32,33,34], encompassing 117,841 individuals, of whom 50,842 were diagnosed with *H. pylori*. All included studies were conducted in Russia.

In most studies, *H. pylori* infection was diagnosed using validated techniques, primarily IgG quantification for *H. pylori* (serological testing for *H. pylori*) (*n* = 9) [17,21,22,23,24,25,26,27,33]. Eight of the studies employed a ^13^C-urea breath test [10,15,20,28,29,30,31,32]. Three studies employed uncommon identification approaches [18,19,34]. Fourteen studies scored seven or higher on the NOS, indicating a low risk of bias [10,15,17,20,23,24,26,27,28,29,30,31,33,34].

The weighted mean age across available studies was calculated to provide an overview of the participant demographics. The mean weighted age of participants across the studies was 43.71 ± 16.23, reflecting a predominantly middle-aged population.

### 3.2. The Prevalence of H. pylori in Russia

**Figure 2:**
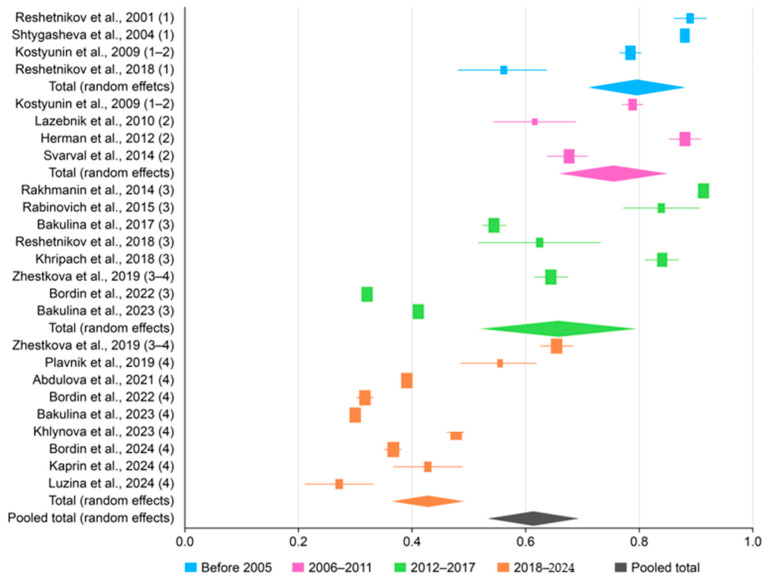
A forest plot showing the pooled prevalence of *H. pylori* by period, where dark blue represents studies before 2005; pink represents studies in 2006–2011; green represents studies in 2012–2017; orange represents studies in 2018–2024; and gray represents the pooled total [10,15,17,18,19,20,21,22,23,24,25,26,27,28,29,30,31,32,33,34].

**Figure 3:**
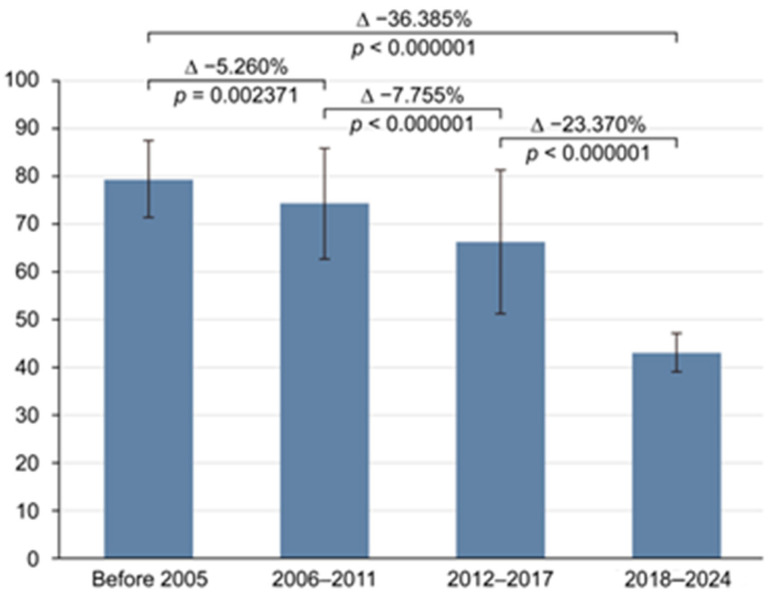
A histogram representing the dynamics of *H. pylori* infection in the Russian population.

**Figure 4:**
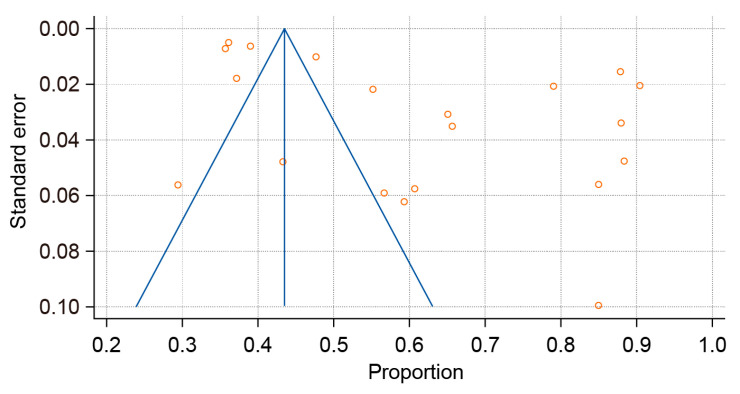
A funnel plot estimating the likelihood of publication bias when calculating the proportion of *H. pylori* patients.

The overall pooled prevalence of *H. pylori* infection among the adult population of Russia over the 30-year study period (1994–2024) was 62.847% (95% CI: 55.101–70.274; Figure 2) with Tau^2^ = 438.59 and Q statistics = 1213.6. A breakdown by study period revealed prevalences of 79.334% (95% CI: 71.925–85.866) before 2005; 74.074% (95% CI: 62.468–84.179) between 2006 and 2011; 66.319% (95% CI: 50.734–80.27) between 2012 and 2017; and 42.949% (95% CI: 38.963–46.982) after 2018. Due to significant heterogeneity (I^2^ > 98% for all periods; *p* < 0.0001), a random-effects model was applied. A histogram was created to visualize the significant decline in *H. pylori* prevalence over time (Figure 3). Publication bias was assessed using a funnel plot (Figure 4), the Begg–Mazumdar test, and Egger’s test. Egger’s regression intercept was 21.4023 (95% CI: 7.4817–35.3230), with a *p*-Value of 0.0046 (indicating possible small-study effect), and Begg–Mazumdar’s test revealed a *p*-Value of 0.05263, suggesting no strong evidence of publication bias, though some uncertainty remains due to the incline in studies to the right on the funnel plot.

### 3.3. Meta-Regression

**Figure 5:**
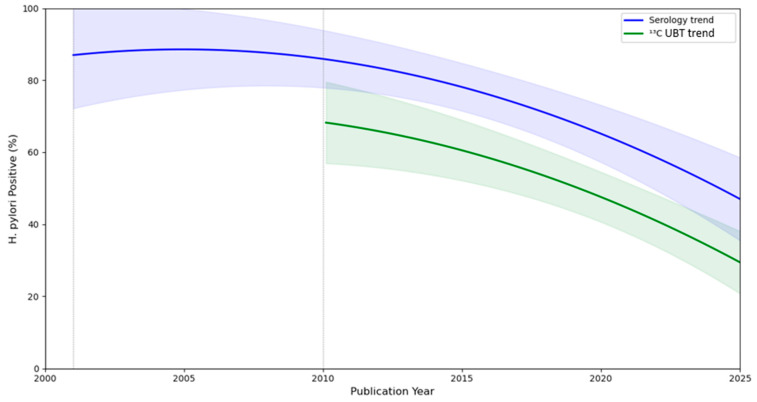
Meta-regression analysis of *H. pylori* prevalence over time by diagnostic method.

A meta-regression analysis was carried out in Python to visually summarize the relationship between the year of publication and the proportion of *H. pylori*-positive patients across multiple studies (Figure 5). The meta-regression line and 95% CI were used to provide a quantitative estimate of the association to determine whether *H. pylori* prevalence changes significantly over time and the methodology used for determination. The trend in the prevalence of *H. pylori* over time was statistically significant, indicating a decrease (regression coefficient for year: −3.773%, *p* < 0.001). The *p*-Value, calculated using a nonlinear model, represents the probability of observing such a strong relationship by chance if no true relationship existed. Diagnostic method was selected as a covariate. A *p*-Value < 0.001 provides strong evidence to reject the null hypothesis and supports the conclusion that the prevalence of *H. pylori* has significantly decreased over the study period.

### 3.4. Subgroup Analysis

We conducted sub-analyses of studies carried out in major cities across Russia, each including three or more studies with comprehensive descriptions of patient populations. First, we evaluated the pooled prevalence of *H. pylori* in Moscow, analyzing seven studies [15,20,21,22,26,32,33]. Out of 7581 patients, 5901 were identified as infected, yielding a pooled prevalence of 66.534% (95% CI: 42.097–86.989). Next, we assessed the pooled prevalence in Saint Petersburg [15,23,30], where 17,203 of 44,048 patients were found to be infected, corresponding to a prevalence of 50.598% (95% CI: 30.422–70.673). Finally, we analyzed data from Novosibirsk [15,17,25], where 557 of 722 patients tested positive for *H. pylori*, yielding a prevalence of 72.251% (95% CI: 46.277–91.990). These findings provide a deeper understanding of the distribution of *H. pylori* infection across diverse geographic areas in Russia.

Additionally, we conducted a subgroup analysis to evaluate the pooled prevalence of *H. pylori* infection, stratified by diagnostic method (Table 2). Over the 30-year study period (1994–2024), the prevalence of *H. pylori* positivity was 45.143% (95% CI: 41.390–48.923) when using the ^13^C-urea breath test and 75.806% (95% CI: 64.213–85.742) with serological testing. Moreover, we analyzed the prevalence rates by time period and method of testing. Until 2018, a high seropositivity of *H. pylori* was observed, exceeding 70% with serological testing for *H. pylori*. Before 2005, seropositivity was 74.625% (95% CI: 40.693–96.927) [17,26]; in 2005–2011, it was 77.533% (95% CI: 52.005–95.139) [21,23]; and in 2012–2017, it reached 78.677% (95% CI: 61.839–86.248) [22,24,25,26,27]. However, from 2018 to 2024, a significant decline in seropositivity to 54.650% (95% CI: 32.892–75.514) [27,33] was noted. A similar trend was observed with the ^13^C-urea breath test. In 2005–2011, the prevalence of *H. pylori* was 60.667% (95% CI: 54.889–66.231) [20], while in 2012–2017, it decreased to 45.404% (95% CI: 27.197–64.272) [14,30], and from 2018 to 2024, it declined further to 41.097% (95% CI: 37.038–45.218) (Table 2) [10,28,29,30,31,32].

### 3.5. Sensitivity Analysis

A sensitivity analysis based on NOS scores addressed heterogeneity by stratifying the studies by methodological quality. Fourteen studies with NOS scores of seven or higher [10,15,17,20,23,24,26,27,28,29,30,31,33,34] reported a pooled prevalence of 56.674% (95% CI: 51.962–61.327). Meanwhile, seven other studies with NOS scores lower than seven revealed a pooled prevalence of 75.074% (95% CI: 55.256–90.534). The lower prevalence in high-quality studies compared to lower-quality studies suggests that methodological quality may influence the reported prevalence of *H. pylori* infection. The higher prevalence in lower-quality studies could be due to biases, such as selection bias, measurement error, or confounding, which are more likely in studies with poorer methodological rigor. In addition, the observed difference between higher- and lower-quality studies may be further compounded by a temporal interaction, as many of the methodologically weaker studies were also conducted in earlier periods.

## 4. Discussion

*H. pylori* is a widely prevalent pathogen that plays a significant role in the development of various gastroduodenal diseases, including GC [1,3,35]. The transmission of *H. pylori* occurs directly from person to person, without the involvement of vectors or intermediate hosts [35,36]. Three main transmission routes are generally recognized: oral–oral, fecal–oral, and iatrogenic (during endoscopic procedures, pH monitoring probe, etc.) [37,38]. Researchers from various countries have demonstrated a clear correlation between the prevalence of *H. pylori* infection and factors such as the overall economic development of a country, standard of living, education level, adherence to sanitary and hygienic norms, per capita income, population density, and availability of adequate living conditions [36,38]. According to recent systematic reviews, approximately 48.6% (95% CI: 43.8–53.5) of the global adult population is infected with *H. pylori*, while the prevalence among children and adolescents is around 32.3% (95% CI: 27.3–37.8) [7,39]. The highest prevalences of *H. pylori* infection are observed in developing countries, exceeding 70% of the population [7].

### 4.1. Main Findings

Russia, as a transcontinental country spanning Europe and Asia, exhibits variability in the prevalence of *H. pylori* infection across its regions. This meta-analysis, which combined the results of 20 studies, demonstrated that the overall prevalence of *H. pylori* in Russia over the analyzed period (1994–2024) was 62.847% (95% CI: 55.101–70.274). To evaluate the trends in prevalence, the analyzed pool of studies was categorized into four distinct time intervals. In the initial period (prior to 2005), the prevalence of *H. pylori* infection was 79.334% (95% CI: 71.925–85.866). This figure declined to 74.074% (95% CI: 62.468–84.179) during the 2006–2011 interval, followed by a further reduction to 66.319% (95% CI: 50.734–80.270) between 2012 and 2017. By the final period (2018–2024), the prevalence had dropped significantly to 42.949% (95% CI: 38.963–46.982). Fisher’s exact test showed significant differences across periods (*p* < 0.000001), which was further confirmed by a meta-regression analysis of the entire pool of studies in chronological order (*p* < 0.00001). Thus, a trend toward a reduction in the prevalence of *H. pylori* infection is evident in Russia, consistent with global practices. A meta-analysis (2014–2023) found that *H. pylori* infection prevalence in China dropped to 42.8%, showing a clear decline over the past decade. Our results are similar: we observed a steady decrease from 79.334% before 2005 to 42.949% after 2018. Both studies highlight a significant downward trend, though our data show a sharper decline, possibly due to differences in regions, populations, or diagnostic methods [40].

The improvement in the epidemiological structure in Russia is associated with various factors. Firstly, over the past few decades, there has been a decrease in overcrowding, as well as improvements in sanitary and hygienic conditions. Secondly, the decrease in the infection rate of the population is undoubtedly linked to the proactive stance of professional medical communities, the integration of clinical guidelines into medical practice, increased accessibility to validated diagnostic methods for *H. pylori* infection (particularly the ^13^C-urea breath test), and the enhancement in the quality and quantity of prescribed eradication therapy (ET). It is worth noting that, according to a pharmacoepidemiologic retrospective analysis (1398 outpatient records) conducted 20 years ago (2004–2005), rational ET was administered in only 18% of cases, with the main errors being irrational drug combinations (34%), monotherapy (30%), and inadequate dosing of appropriately chosen medications (4.3%) [41]. Since the Russian Gastroenterological Association introduced the first clinical guidelines for the diagnosis and treatment of *H. pylori* infection in adults in 2012 [42], this negative trend has been reversed, as reflected in the current epidemiological structure [43]. However, barriers to eradication remain, including rising antibiotic resistance [44], limited access to molecular and urea breath testing in some regions, and challenges with patient adherence to multiday regimens.

Additionally, the European Registry on the management of *H. pylori* infection (Hp-EuReg), an observational multicenter prospective study initiated by the European *Helicobacter* and Microbiota Study Group, has played a crucial role in analyzing and improving the management of *H. pylori* infection in Russia [45,46]. An analysis of Hp-EuReg data from Moscow revealed positive changes since 2013: a significant increase in the use of 14-day regimens (which have predominated since 2016), the addition of bismuth to triple therapy in most cases, and more frequent use of the ^13^C-urea breath test to assess eradication efficacy [47]. In recent years, it has been demonstrated that 14-day quadruple-therapy regimens (proton pump inhibitor + metronidazole + tetracycline + bismuth and proton pump inhibitor + clarithromycin + amoxicillin + bismuth), that have been used frequently in Russia, achieve a high level of eradication according to meta-analyses [48,49]. The most recent updated version of Russian *H. pylori* treatment guidelines also support these findings [50]. A positive reflection of these developments is the trend toward a reduction in GC incidence in Russia, which is undoubtedly associated with the decline in *H. pylori* prevalence [12,51].

### 4.2. Limitations

Our study has several limitations. First, only 20 studies, spanning 30 years, could be included, reflecting the scarcity of Russian epidemiological data. This results in high heterogeneity in data synthesis, although significant publication bias was ruled out based on the Begg–Mazumdar test. Second, the diagnostic methods used in the included studies varied, ranging from the ^13^C-urea breath test [10,15,20,28,29,30,31,32] to serological assays [17,21,22,23,24,25,26,27,33]. We decided to combine the results of these methodologies to calculate the overall prevalence and infection dynamics, but we also conducted subgroup analyses separately for each diagnostic method. Third, in two studies [15,33], the adult population consisted of healthcare workers; however, we believe their inclusion did not materially bias the pooled estimates, given the random sampling and the absence of pre-diagnostic data on existing gastroduodenal diseases.

In light of the high heterogeneity observed across studies (I^2^ > 98%), a narrative synthesis is warranted to complement the meta-analytic results. To systematically explore these differences, we created Table 3, which summarizes population type (general, clinical, or occupational) and detects the risk of bias contributed by these factors. The variation in *H. pylori* prevalence may be partially explained by differences in the study populations (Table 3) and regional socioeconomic conditions. For example, studies conducted in urban centers like Moscow and Saint Petersburg often involved more access to modern diagnostic tools such as the ^13^C-urea breath test, which tends to yield lower prevalence estimates than serological testing. Additionally, urban populations may have benefited earlier from improvements in sanitation and access to medical care, potentially contributing to the lower rates of infection observed in more recent studies from these regions.

The use of different diagnostic methods is another significant contributor to heterogeneity. Serological testing, which was more commonly used in earlier studies, can detect past as well as current infections, often resulting in higher prevalence estimates. In contrast, the 13C-urea breath test is more specific to active infections and became more widely available in later years. This methodological shift may partly explain the downward trend observed across the study periods.

Study quality also played a role. As shown in the sensitivity analysis, studies with lower methodological quality tended to report higher prevalence. This could be due to selection bias, outdated diagnostic approaches, or less representative populations. Furthermore, the inclusion of healthcare workers in a few studies may have introduced additional variation, although these populations were included based on randomized recruitment and represented a specific adult cohort.

Taken together, these factors indicate a clear downward trend, but the magnitude of the decline should be interpreted in light of differences in study design, setting, diagnostic method, population, and time period.

Although high heterogeneity was explored, the use of a random-effects model was methodologically appropriate, as it accounts for between-study variability and reflects the diversity of populations, diagnostic approaches, and study designs. Furthermore, subgroup and meta-regression analyses were applied to identify and quantify temporal trends, enhancing the robustness of our findings.

Despite these limitations, this meta-analysis represents the first comprehensive work summarizing the results of epidemiological studies assessing the prevalence of *H. pylori* infection in Russia and is the first to document, with meta-analytic and meta-regression techniques, a clear chronological decline over three decades.

## 5. Conclusions

In conclusion, this meta-analysis demonstrates a gradual decline in the prevalence of *H. pylori* infection in Russia. However, the prevalence among the adult population remains relatively high, underscoring the need to continue programs for the early diagnosis of *H. pylori* infection and subsequent eradication therapy to reduce the risk of associated diseases, including GC.

## Figures and Tables

**Table 1:** Characteristics of included studies.

Study, Year	Methodology of *H. pylori* Diagnostic	Geographical Location	Total Number of Patients, n	Total Number of Patients with *H. pylori*	Mean Age, Years	Period of Inquiry	NOS Criteria Score
Reshetnikov et al., 2001 [17]	Serology	Novosibirsk	438	387	44.36 ± 16.38	1994–1995	8
Shtygasheva et al., 2004 [18]	Four methods (morphological, urease breath test, polymerase chain reaction in biopsy, serological testing)	Khakassia Republic	4217	3625	not available	2004	3
Kostyunin et al., 2009 [19]	Examination of gastrobiopathology (histologically and cytologically)	Irkutsk Region	2324	1836	44.36 ± 0.33	2001–2006	6
Lazebnik et al., 2010 [20]	^13^C-urea breath test	Moscow	300	182	56.6 ± 15.3	2006	7
Herman et al., 2012 [21]	Serology	Moscow	863	759	not available	2011	6
Rakhmanin et al., 2014 [22]	Serology	Moscow	2414	2182	not available	2013	5
Svarval et al., 2014 [23]	Serology	Saint Petersburg	1057	688	not available	2007–2011	7
Rabinovich et al., 2015 [24]	Serology	Ural Federal District	100	85	58.6 ± 15	2015	7
Bakulina et al., 2017 [15]	^13^C-urea breath test	Multicenter Research	2098 (of these, 127 patients in Moscow; 74 in Kazan; 448 in Saint Petersburg; 26 in Novosibirsk; and 10 in other cities)	1157 (of these, 61 patients in Moscow; 38 in Kazan; 227 in Saint Petersburg; 17 in Novosibirsk; and 10 in other cities)	48 ± 13.5	2016–2017	7
Reshetnikov et al., 2018 [25]	Serology	Novosibirsk	168	97	not available	2003–2005	5
			90	56		2013–2015	
Khripach et al., 2018 [26]	Serology	Moscow	319	271	42 ± 14.07	2017	7
Zhestkova et al., 2019 [27]	Serology	Ryazan Region	809	531	57.38 ± 11.63	2017–2018	8
Plavnik et al., 2019 [28]	^13^C-urea breath test	Kazan and Moscow	286	162	38 ± 12.5	2019	7
Abdulova et al., 2021 [29]	^13^C-urea breath test	Multicenter Research	26,127	10,190	42.9 ± 17.8	2019–2020	7
Bordin et al., 2022 [10]	^13^C-urea breath test	Multicenter Research	10,225	3669	43.65 ± 15.5	2017	9
			9650	3431		2019	
Bakulina et al., 2023 [30]	^13^C-urea breath test	Saint Petersburg	8553	3537	43.76 ± 15.73	2015–2017	8
			33,990	11,821		2018–2023	
Khlynova et al., 2023 [31]	^13^C-urea breath test	Ural Federal District	9939	4733	42.95 ± 17.77	2018–2022	7
Bordin et al., 2024 [32]	^13^C-urea breath test	Moscow	3124	1162	not available	2022	6
Kaprin et al., 2024 [33]	Serology	Moscow	434	188	48.5 ± 0.6	2024	7
Luzina et al., 2024 [34]	Detection of *H. pylori* antigen in feces by single-stage immunochromatographic analysis	Zabaykalsky Krai	316	93	46.83 ± 14.66	2019–2023	7

**Table 2:** Rates of positive detection for *H. pylori* by diagnostic method and time period.

Diagnostic Method	Before 2005, %	2005–2011, %	2012–2017, %	2018–2024, %
Serology	74.625 (95% CI: 40.693–96.927)	77.533 (95% CI: 52.005–95.139)	78.677 (95% CI: 61.839–86.248)	54.650 (95% CI: 32.892–75.514)
^13^C-urea breath test	No data	60.667 (95% CI: 54.889–66.231)	45.404 (95% CI: 27.197–64.272)	41.097 (95% CI: 37.038–45.218)

**Table 3:** Overview of sociodemographic context and risk of bias assessment.

Study	Sampling Population	ROBINS-I Risk of Bias (Participant Selection) [52]
Reshetnikov et al., 2001 [17]	General population	Low risk
Shtygasheva et al., 2004 [18]	Multiple population groups	Moderate risk
Kostyunin et al., 2009 [19]	Gastroenterology patients	High risk
Lazebnik et al., 2010 [20]	General population	Low risk
Herman et al., 2012 [21]	General population	Low risk
Rakhmanin et al., 2014 [22]	General population	Low risk
Svarval et al., 2014 [23]	Gastroenterology patients	High risk
Rabinovich et al., 2015 [24]	Multiple population groups	Moderate risk
Bakulina et al., 2017 [15]	Healthcare workers	High risk
Reshetnikov et al., 2018 [25]	General population	Low risk
Khripach et al., 2018 [26]	General population	Low risk
Zhestkova et al., 2019 [27]	Multiple population groups	Moderate risk
Plavnik et al., 2019 [28]	General population	Low risk
Abdulova et al., 2021 [29]	General population	Low risk
Bordin et al., 2022 [10]	General population	Low risk
Bakulina et al., 2023 [30]	General population	Low risk
Khlynova et al., 2023 [31]	Gastroenterology patients	High risk
Bordin et al., 2024 [32]	General population	Low risk
Kaprin et al., 2024 [33]	Healthcare workers	High risk
Luzina et al., 2024 [34]	General population	Low risk

## Data Availability

No new data were created or analyzed in this study. Data sharing is not applicable to this article.

## Supplementary Materials

The following supporting information can be downloaded at https://www.mdpi.com/article/10.3390/epidemiologia6030047/s1, File S1. Completed PRISMA-P checklist. File S2. Completed NOS evaluation.

## Abbreviations

GC	Gastric cancer
PRISMA	Preferred Reporting Items for Systematic Reviews and Meta-Analyses
PROSPERO	International Prospective Register of Systematic Reviews
RSCI	Russian Science Citation Index
NOS	Newcastle–Ottawa scale
*H. Pylori*	*Helicobacter pylori*
CI	Confidence interval
MRSA	Methicillin-resistant *Staphylococcus aureus*
WHO	World Health Organization
IARC	International Agency for Research on Cancer
MALT	Mucosa-associated lymphoid tissue
Hp-EuReg	*Helicobacter pylori* European Registry
ET	Eradication therapy
PPI	Proton pump inhibitor
