# Supplementary material for: The Prevalence of Helicobacter pylori Infection in the Adult Population of Russia: A Systematic Review and Meta-Analysis
Source: Epidemiologia (Basel). 2025 Aug 12;6(3):47. doi: 10.3390/epidemiologia6030047 (PMC12372033; doi:10.3390/epidemiologia6030047)
Supplement: Supplementary file 1 [file epidemiologia-06-00047-s001.zip › File S2. Completed NOS evaluation.pdf]

Study	**Selection (4)**	**Comparability (2)**	**Outcome (3)**	**Total**
Reshetnikov et al. 2001	★★★	★★	★★★	8
Shtygasheva et al., 2004	★	★	★	3
Kostyunin et al., 2009	★★	★★	★★	6
Lazebnik et al. 2010	★★★	★★	★★	7
Herman et al. 2012	★★★★	★★	★	6
Rakhmanin et al. 2014	★★	★★	★	5
Svarval et al. 2014	★★★	★★	★	7
Rabinovich et al. 2015	★★★	★★	★★	7
Bakulina et al. 2017	★★★	★	★★★	7
Reshetnikov et al. 2018	★★	★★	★	5
Khripach et al. 2018	★★★	★★	★★	7
Zhestkova et al. 2019	★★★	★★	★★★	8
Plavnik et al. 2019	★★★	★	★★★	7
Abdulova et al. 2021	★★★★	★	★★	7
Bordin et al. 2022	★★★★	★★	★★★	9
Bakulina et al. 2023	★★★	★★	★★★	8
Khlynova et al. 2023	★★★	★	★★★	7
Bordin et al. 2024	★★	★★	★★	6
Kaprin et al. 2024	★★★★	★	★★	7
Luzina et al., 2024	★★★	★★	★★	7
